# Vestibular function in the temporal and parietal cortex: distinct velocity and inertial processing pathways

**DOI:** 10.3389/fnint.2014.00053

**Published:** 2014-07-04

**Authors:** Jocelyne Ventre-Dominey

**Affiliations:** INSERM U846, Stem Cell and Brain Research Institute, Lyon UniversityBron, France

**Keywords:** vestibular, parieto-temporal cortex, MST, spatial representation, self-referential, egomotion

## Abstract

A number of behavioral and neuroimaging studies have reported converging data in favor of a cortical network for vestibular function, distributed between the temporo-parietal cortex and the prefrontal cortex in the primate. In this review, we focus on the role of the cerebral cortex in visuo-vestibular integration including the motion sensitive temporo-occipital areas i.e., the middle superior temporal area (MST) and the parietal cortex. Indeed, these two neighboring cortical regions, though they both receive combined vestibular and visual information, have distinct implications in vestibular function. In sum, this review of the literature leads to the idea of two separate cortical vestibular sub-systems forming (1) a velocity pathway including MST and direct descending pathways on vestibular nuclei. As it receives well-defined visual and vestibular velocity signals, this pathway is likely involved in heading perception and rapid top-down regulation of eye/head coordination and (2) an inertial processing pathway involving the parietal cortex in connection with the subcortical vestibular nuclei complex responsible for velocity storage integration. This vestibular cortical pathway would be implicated in high-order multimodal integration and cognitive functions, including world space and self-referential processing.

## INTRODUCTION

Since the early clinical observations suggestive of a cortical role in vestibular function (Carmichael et al., 1954; Penfield and Jasper, 1954), empirical data on the underpinnings of such a cortical vestibular integration have been provided only in the 1980s, by behavioral and electrophysiological recording experiments conducted in animals. Thus, by neural unit recordings, discharges of neurons located in the visual associative cortex were observed during body rotations in cat (Becker et al., 1979; Deecke et al., 1979; Mergner, 1979; Vanni-Mercier and Magnin, 1982a,b) and in monkey (Kawano et al., 1980, 1984; Kawano and Sasaki, 1984; Akbarian et al., 1988; Grusser et al., 1990a,b). More precisely, visual tracking neurons were found in monkey to receive vestibular information in cortical sites located in the associative parietal and temporal cortex (Kawano et al., 1980, 1984; Kawano and Sasaki, 1984) and in the retro-insular cortex (Grusser et al., 1990a,b). In our early work studying effects of cortical lesions in the cat (Ventre, 1985a,b), we described a parcellation of visuo-vestibular areas distributed in the suprasylvian (SS) cortex divided into two regions: the middle SS gyrus (area 7) preferentially involved in vestibularly driven ocular responses and the lateral SS sulcus implicated in optokinetic ocular responses. Similarly, a pattern of visual extrastriate areas localized in the superior temporal sulcus in monkey and described as MT (middle temporal) and MST (middle superior temporal) areas were found to be activated during visual motion as well as during visuo-vestibular interactions (Komatsu and Wurtz, 1988a,b; Newsome et al., 1988), thereby suggesting in primate similar visual and vestibular cortical organization as in the cat (Tusa et al., 1989; Ventre, 1985a,b). Indeed, based on recent data on neuronal receptive field properties and visual behavior (Lomber and Payne, 2004), this lateral suprasylvian sulcus (area PMLS) is equated to the visual areas located in the middle temporal sulcus in macaque monkey.

**FIGURE 1:**
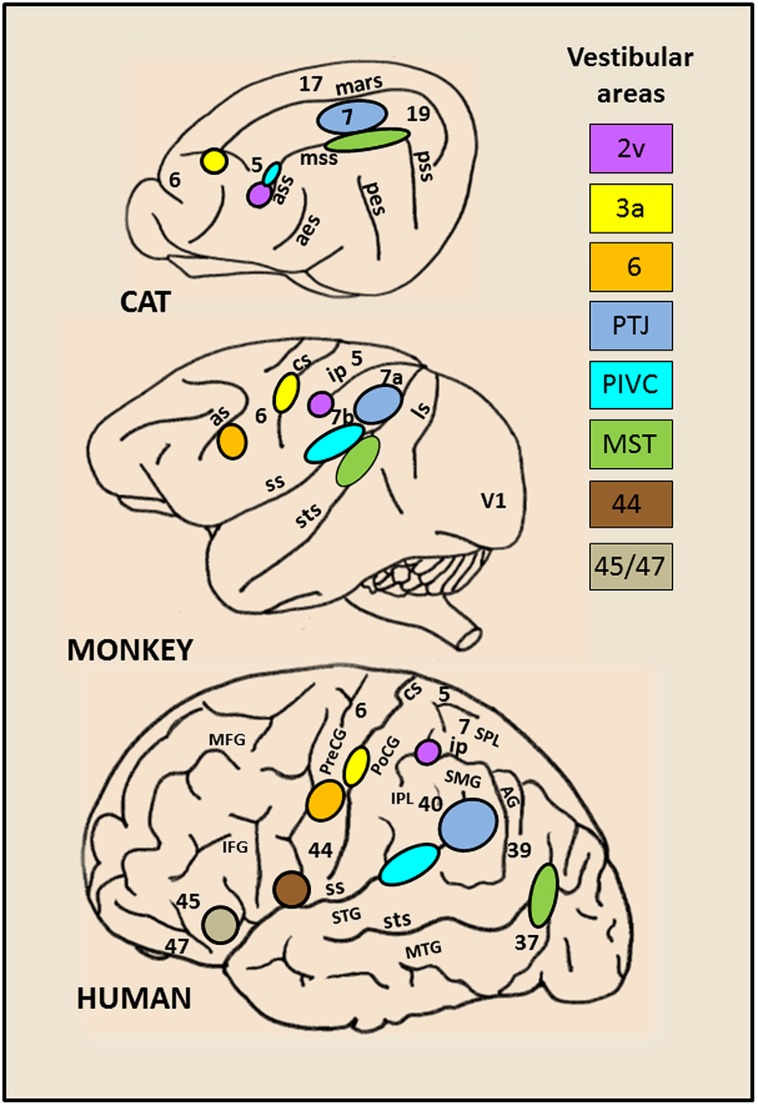
**Schematic brain representations illustrating the topography of the vestibular cortical fields as they have been experimentally identified in cat, monkey, and human.** The numbers on the cortex indicate the architectonically defined Brodman’s areas (based on (Talairach and Tournoux, 1988). In the right panel are listed the vestibular sites with their localization in the cortical regions. In cat: the marginal sulcus (mars), the anterior (ass), middle (mss) and posterior (pss) suprasylvian sulci, the anterior (aes) and posterior (pes) ectosylvian sulci. In primate (monkey and human): the lunate sulcus (ls), the superior temporal sulcus (sts), the sylvian scissure (ss), the intraparietal sulcus (ip), the central sulcus (cs) and the arcuate sulcus (as), the superior (STS) and middle (MTG) temporal gyri, the angular gyrus (AG), the supramarginal gyrus (SMG), the superior (SPL) and inferior (IPL) parietal lobes, the postcentral (PoCG) and precentral (PreCG) gyri, the middle (MFG) and inferior (IFG) frontal gyri.

By combining lesions and tracer injections (Ventre and Faugier-Grimaud, 1986, 1989; Faugier-Grimaud and Ventre, 1989) or electrophysiology and tracer injections (Akbarian et al., 1994, 1993) evidence has been provided in monkey of a larger vestibular network distributed between the parieto-temporal cortex, the retro-insular and the prefrontal cortex and directly connected to the vestibular nuclei complex. Only recently, over the last two decades, the advanced neuroimaging techniques have given access to the investigation of human brain activation elicited during either caloric ear irrigation or galvanic vestibular stimulation (Bottini et al., 1994, 2001; Bucher et al., 1997; Vitte et al., 1996; Lobel et al., 1998; Fasold et al., 2002; Dieterich et al., 2003; Emri et al., 2003; Naito et al., 2003; Stephan et al., 2005; Eickhoff et al., 2006; Lopez et al., 2012; Zu Eulenburg et al., 2012). These authors demonstrated in healthy subjects that such vestibular stimulations triggered activity in distributed cortical areas including the posterior temporo-parietal and retro-insular cortex, the intraparietal sulcus (homologue to area 2v), the somatosensory area 3 as well as rostrally several frontal regions (middle and inferior frontal gyri) and the anterior cingulate cortex. Interestingly, there is a striking homology between the vestibular cortical networks described in monkey and in man (see for review,Fukushima, 1997; Brandt and Dieterich, 1999; Lopez and Blanke, 2011). **Figure 1** illustrates the inter-species organization and growing complexity of the vestibular cortical fields. While convincing evidence is now provided for a link between vestibular function and cortical processes, the exact roles of these various cortical sites topographically similar in animal and human, remain obscure. Recent studies suggest a role of some of these cortical vestibular sites in cognitive processes relying on vestibular integration i.e. spatial representation and self-consciousness (see for review, Lopez and Blanke, 2011).

On the basis of the subcortical organization of the vestibular function largely described in the past (Robinson, 1977; Raphan et al., 1979; Cohen et al., 1981; Raphan and Cohen, 1985), the vestibulo-ocular responses rely on two cooperating kinematic processes: (1) the direct process is responsible for the gain of the ocular responses that will trigger ocular responses to rapidly compensate for head motion and (2) the indirect process is responsible for a low-frequency kinematic component insuring a multimodal integration of the vestibular signals for the purpose of storage and updating of spatial coordinates (Wearne et al., 1997, 1998; Cohen et al., 1999; Raphan and Cohen, 2002; Leigh, 2006). Such a dual path organization of the vestibular system is also reflected in vestibular evoked control of goal-directed arm movements (Bresciani et al., 2002, 2005).

In the following review of the literature, we will attempt to draw a parallel between such a dual organization of the vestibular function as demonstrated in subcortical regions, and the organization of the temporo- parietal cortex. Thus, we hypothesize that the cortical integration of vestibular information is organized as a twofold system of pathways originating from the temporal and parietal cortices which respectively mediate vestibularly driven velocity and inertial signals. As the cortical properties have been extensively investigated in human and non-human primate, we will mainly refer in the following to the studies describing the cortical processes linked to ego-motion in these species. However, the inter-species similarities described above demonstrate the functional coherence and evolutionary continuity that emphasize the physiological foundations for visual and vestibular interactions in the cerebral cortex especially in the parietal and temporal lobes.

## IS THE TEMPORAL CORTEX INVOLVED IN A VELOCITY PATHWAY?

A perceived displacement of the visual surroundings can be triggered by the retinal slip of the visual environment or by head/body motion. In order to differentiate between the motion of the visual surrounding versus self-motion, the central nervous system must integrate multimodal signals including visual and vestibular signals in order to extract the origin and direction of the perceived movement. As mentioned above, cortical visual and vestibular interaction has been first suggested in the visual lateral suprasylvian cortex in cat (Vanni-Mercier and Magnin, 1982a,b; Ventre, 1985a,b; Rauschecker et al., 1987; Tusa et al., 1989) and in the middle temporal sulcus in macaque monkey (Dursteler and Wurtz, 1988; Komatsu and Wurtz, 1988a,b). These visual temporal cortical areas called MT (middle temporal) and MST (middle superior temporal) have a critical role in visual motion processes linked to smooth pursuit, heading perception and optokinetic-related information, all involving velocity signals triggered during ego-motion in monkey. In the following, we will see how the visual and vestibular signals are processed in the temporal cortex (MST) to provide velocity information about ego-motion. The main findings leading to the idea of a velocity pathway in the temporal cortical region will be developed in the two next sections successively for the integration of visual (visual motion) and vestibular (body motion) kinetic inputs.

### VISUAL COMPONENT OF SELF-MOTION ANALYSIS

Monkey extrastriate visual areas including MT and MST have been largely investigated first in visual motion processing (Zeki, 1974; Maunsell and Van Essen, 1983; Newsome et al., 1985; Desimone and Ungerleider, 1986). Their majority of cells responsive to moving visual stimuli is linked to smooth pursuit (SP) in a preferred direction but differed in the size of their receptive fields, with MST having larger receptive fields (Zeki, 1974; Komatsu and Wurtz, 1988a,b, 1989). These extrastriate visual areas have been first described for their role in pursuit and compensatory eye movement generation due to visual field displacements and more recently they have been implicated in self-motion and heading perception. In the context of self-motion, the most interesting units are in MST, the pursuit cells preferentially activated during moving background (Erickson and Dow, 1989; Inaba et al., 2007). Such pursuit cells have opposite preferred direction for pursuit and visual motion leading to a synergistic response during pursuit in the light (Erickson and Dow, 1989). Interestingly as suggested by Komatsu and Wurtz (1988b), such a synergistic response of MST neurons might be to increase the pursuit response of these cells to compensate for the optokinetic nystagmus. Even though suggested by these electrophysiological works in monkey, the idea of a role of MST in visuo-vestibular function in the primate will only clearly emerge from focal lesions studies. Thus, Dursteler and Wurtz (1988) demonstrated that chemical lesions in MST can induce twofold deficits in optokinetic nystagmus (OKN) (1) a reduction in the slow OKN build-up related to a directional pursuit deficit toward the lesioned side and (2) a reduction in the fast OKN build-up related to a retinal deficit with no specific directional preponderance. In accordance with our own observations in the cat (Ventre, 1985a,b), these findings were the first demonstration in monkey of the contribution of extrastriate cortex, including MST on OKN generation. In humans, OKN deficits described with a reduction of ipsiversive slow phase velocity have been reported first in large cortical lesions, including parietal cortex (Carmichael et al., 1954; Smith and Cogan, 1959) and in a subset of extrastriate cortical areas near the temporo-parieto-occipital region (Thurston et al., 1988; Morrow and Sharpe, 1990; Barton et al., 1996; Heide et al., 1996, Lekwuwa and Barnes, 1996). Taken together, these observations argue in favor of a role of MST area in computation of velocity signals issued from moving surrounding objects and used to produce pursuit and compensatory eye movements like OKN.

**FIGURE 2:**
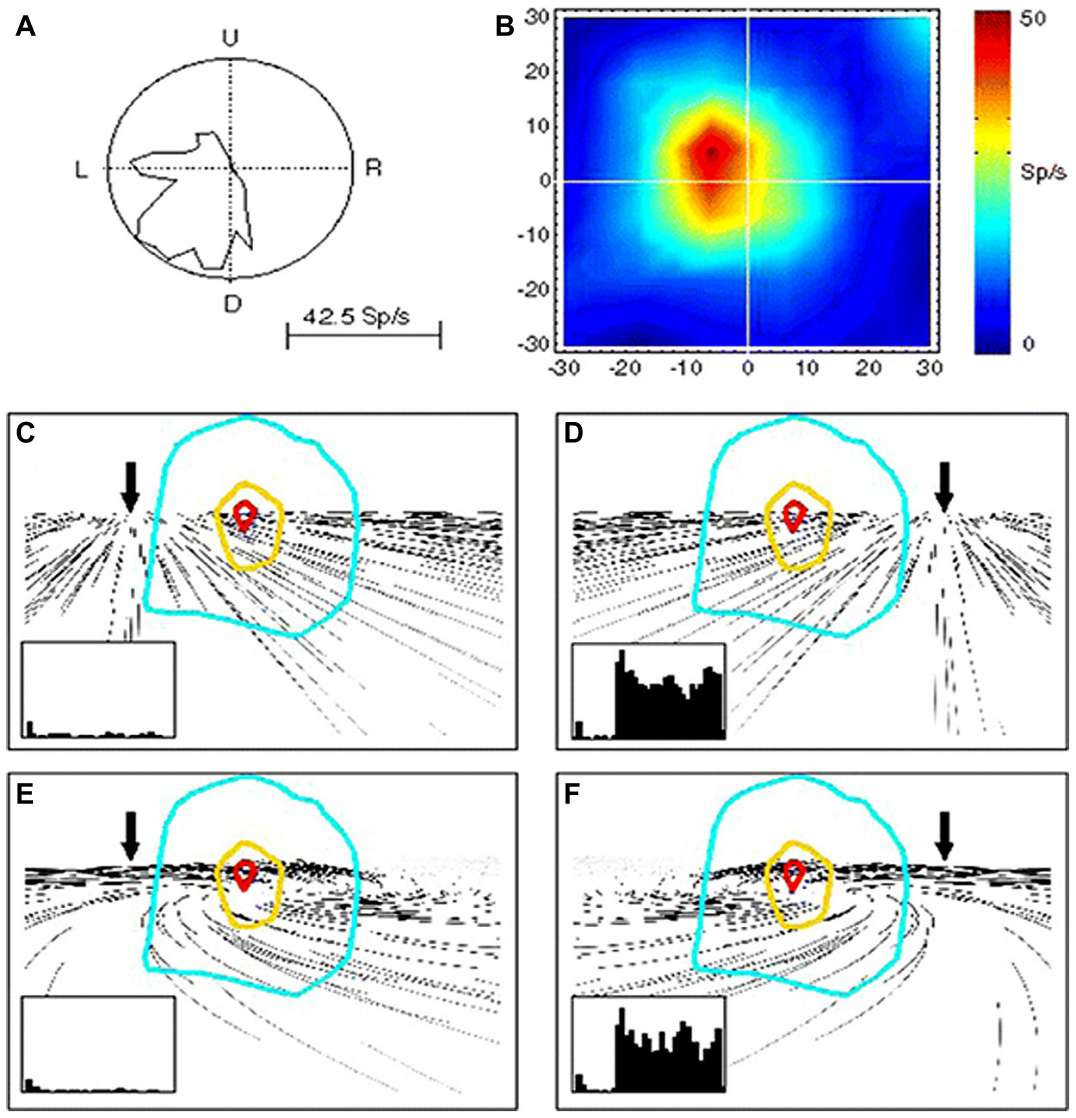
**Responses of a single MST neuron to unidirectional motion and optic flow. (A)** Polar plot of the directional selectivity to left and downward motion. **(B)** Receptive field (RF) characterized by moving luminant bars to the left. **(C–F)** RF outlines on the visual flow stimuli reproducing leftward **(C,E)** and rightward **(D,F)** heading with **(E,F)** and without **(C,D)** eye movements. In the insets are shown the neuron responses in each condition. Note that the neurons is reliably responding to rightward heading (leftward visual flow) **(D,F)** and not to leftward heading (rightward visual flow) irrespective of the eye-movement-related distortion. Reproduced from Bremmer et al. (2010).

This MST activity in computing velocity signals of visual motion can give rise to perception of self-displacements. By studying the effects of optic flow on the activity of MST neurons, evidence has been provided in monkey of a role in heading perception of this visual area (Page and Duffy, 1999; Shenoy et al., 1999; Gu et al., 2006, 2010, 2012; Fetsch et al., 2010). Indeed, MST neurons are tuned to structured visual patterns in movement either in the fronto-parallell, radial, or expansion/contraction directions (Tanaka and Saito, 1989; Tanaka et al., 1989; Graziano et al., 1994; Fetsch et al., 2007). **Figure 2** shows an example of MST cells discharging to simple and distorted flow fields that simulate self-motion plus an eye movement (Bremmer et al., 2010). Bremmer et al. (2010) demonstrate that these MST neurons are able to compensate for visual distortion and to maintain their tuning response to the visual flow direction corresponding to heading information.

It is likely that MST yields a common cortical process sub-serving heading perception and optokinetic response generation, both elicited by large visual field displacements. Recent works (Cardin and Smith, 2011) suggest that a subset of cortical regions, including MST, the ventral intraparietal area, the medial visual area V6 as well as the cingulate cortex could integrate stereoscopic visual cues into ego-motion information. Neuroimaging using PET or fMRI approaches have demonstrated that human occipito-temporal cortex is engaged in the processing of retinal and extraretinal SP velocity as well as optokinetic signals (Barton et al., 1996; Bucher et al., 1997; Freitag et al., 1998; Petit and Haxby, 1999; Nagel et al., 2008). In sum, MST neurons might contribute to distinguish between external versus self-induced motion. However, such a computation requires that the visual signals about the environment displacement and the vestibular signals about the body motion are integrated in this same cortical region. The next section will show how combined with the visual signals, the vestibular signals about body motion will disambiguate ego-motion from object motion.

### VESTIBULAR COMPONENT OF SELF-MOTION ANALYSIS

**FIGURE 3:**
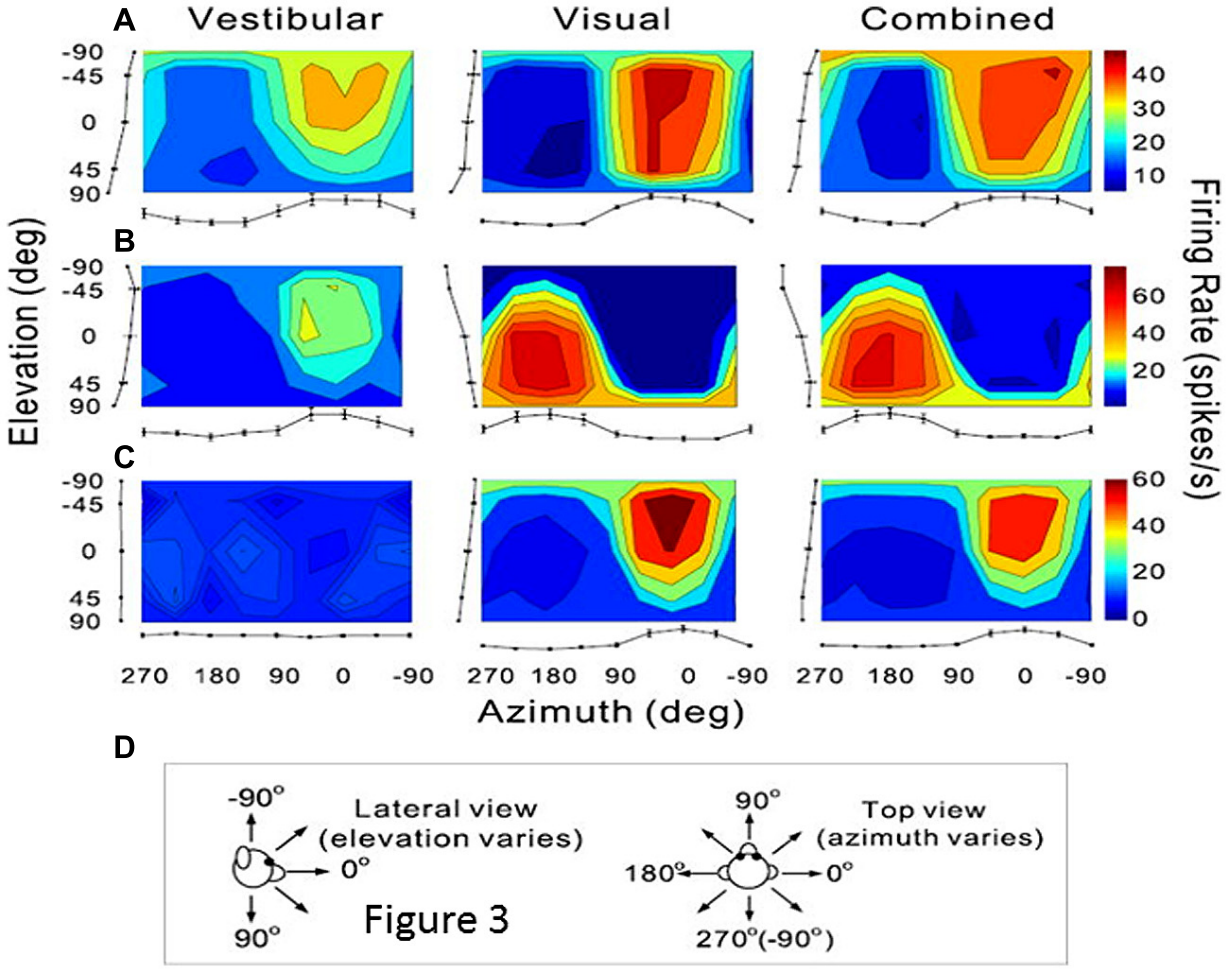
**Vestibular and visual tuning of three MST neurons responding to heading directions defined respectively by inertial motion alone (vestibular), optic flow alone (visual), and congruent combinations of the two cues (combined). (A–C)** Tuning curves showing the firing rates (mean + SEM) represented as the margins of each color map and plotted as a function of the heading direction computed in azimuth and elevation angles. **(D)** Representations of the 3D heading directions in azimuth and elevation angles. **(A)** Neuron with congruent tuning for heading elicited by vestibular and visual cues. **(B)** Neuron with incongruent tuning for heading elicited by vestibular and by visual cues. **(C)** Neuron with selective tuning for visual heading only. Reproduced from Gu et al. (2006).

As previously mentioned, extrastriate visual areas including MST, the medial area V6 and VIP, might participate in the encoding of combined retinal and extraretinal signals including vestibular signals related to head displacements. The most thoroughly investigated is the dorsal part of MST (MSTd) in monkey (Page and Duffy, 1999; Gu et al., 2006, 2010, 2012; Fetsch et al., 2010). Recently a series of electrophysiological experiments in monkey clearly demonstrate that these MSTd neurons were firing during optic flow as well as during vestibular stimulation, hence subtending heading perception in separate reference frames, respectively eye-centered and head-centered (Fetsch et al., 2007; Gu et al., 2007, 2012, Takahashi et al., 2007). In the context of self-motion perception, the notion of an integration of vestibular signals in this extrastriate cortex then follows logically, and is clearly demonstrated via unit recordings in monkey after bilateral labyrinthectomy (Takahashi et al., 2007). In bilaterally labyrinthectomized animals, the MSTd neurons’ firing rate is significantly diminished for physical rotation and translation in the dark, and not in the visual condition. Takahashi et al. (2007) suggested that the vestibular signals in MSTd could compensate for the ambiguous effects of the optic flow information during head movements. Indeed, as illustrated in **Figure 3**, many neurons in MSTd respond during vestibular stimulation in the dark and can display the same or opposite tuning for direction of motion in both visual and vestibular modalities, suggesting multimodal interactions in encoding heading (Bremmer et al., 1999; Gu et al., 2006; Fetsch et al., 2007, 2010). Based on these findings, it is likely that the extrastriate visual area MST contributes to self-motion regulation in coupling visual and vestibular kinetic information in order to compensate for retinal slip and thereby to maintain world stability during ego-motion. If evidence is provided of such MST influence on self-motion, it is not exclusive as visual and vestibular heading encoding has also been found in macaque ventral intraparietal area (VIP; Bremmer et al., 2002; Schlack et al., 2002; Klam and Graf, 2003). However, even though VIP neurons’ discharges have similar selectivity as MSTd, VIP might be more specialized in the detection of targets approaching the face (Colby et al., 1993).

In humans, similar findings have been reported in a cortical network including the homolog of the motion sensitive areas MST i.e., the area BA 37 in the anterior bank of the inferior temporal sulcus (Bense et al., 2001; Huk et al., 2002; Stephan et al., 2005), the medial parietal cortex V6 and the VIP that might support heading perception (Morrone et al., 2000; Baumann and Mattingley, 2010; Cardin and Smith, 2010, 2011; Cardin et al., 2012; Smith et al., 2012; Indovina et al., 2013). However, using functional MRI, Cardin et al. (2012) suggest that only MST is implicated in the extraction of optic flow for computation of heading direction. While MST might provide a representation of heading perception, V6 would rather be concerned with obstacle avoidance during self-motion as inferred by Cardin et al. (2012). By using galvanic stimulation in humans, Smith et al. (2012) have described vestibular inputs in two cortical regions localized in the anterior part of MST and the visual area of cingulate cortex (CSv). Furthermore, by investigating the cortical activation elicited during vection, Brandt et al. (1998) showed that visual-vestibular interactions could take place in the extrastriate visual cortical areas, including the human homolog of MST in BA37. Thus, in order to disambiguate self-motion from object motion, reciprocal excitatory-inhibitory influences would occur within cortical loops including visual extrastriate cortex (MST) and vestibularly activated cortex i.e., temporo-parietal and retro-insular cortex (Brandt et al., 1998; Galati et al., 1999; Kleinschmidt et al., 2002). Brandt et al. (1998) have concluded that the intracortical visuo-vestibular interactions might insure self-motion sensation during body displacements and the opposite during motion of the visual field by respective inhibition of the visual or the vestibular retro-insular cortex.

**FIGURE 4:**
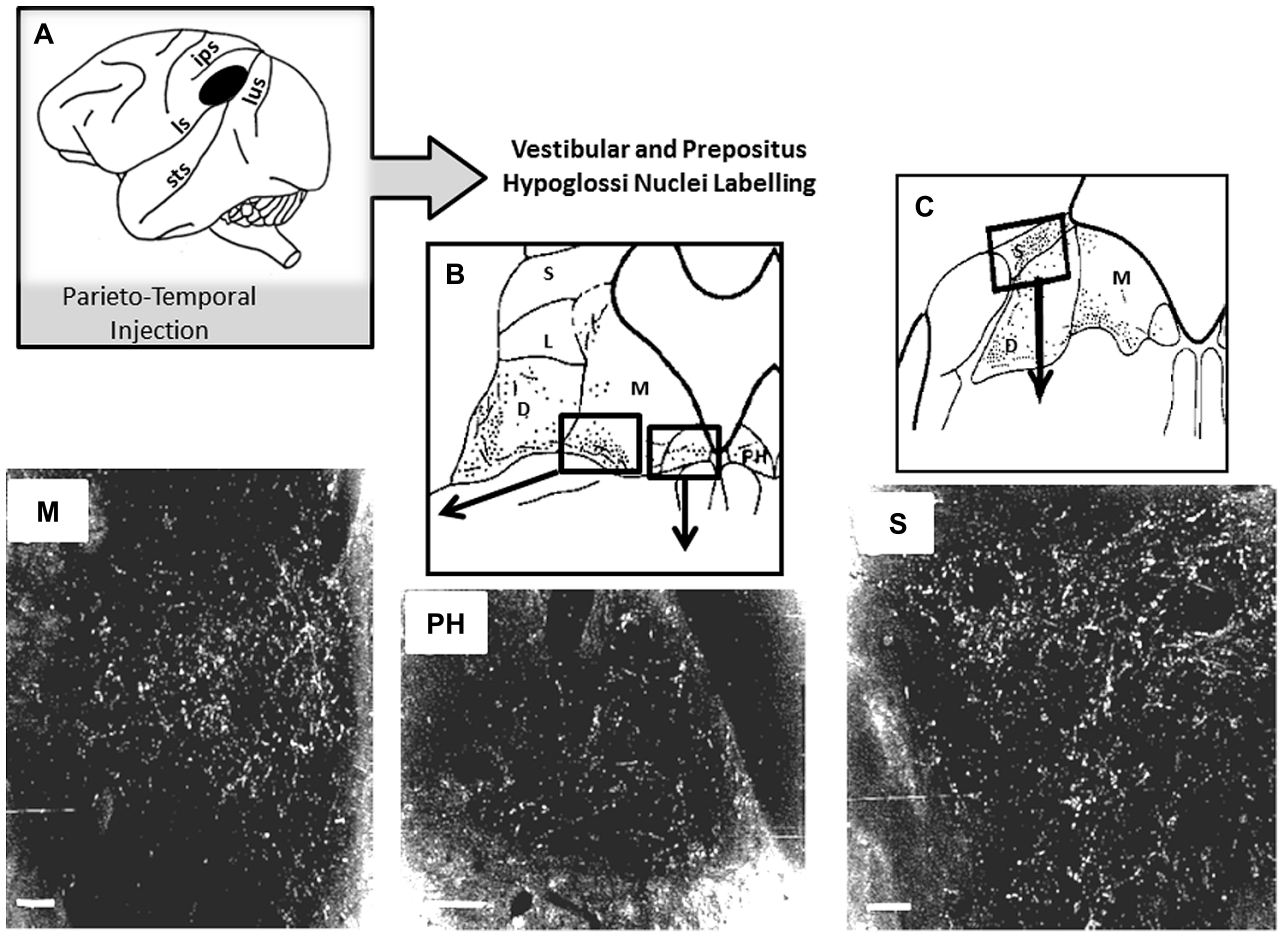
**Horseradish peroxidase (HRP) anterograde labeling in vestibular complex and prepositus hypoglossi in macaque monkey. (A)** Schematic representation of the location of the HRP injection site in the parieto-temporal cortex. **(B,C)** Reconstruction of the labeled zones in the vestibular complex and in the prepositus hypoglossi. In the bottom part, darkfield photomicrographs display bright labeled neurons and fibers in the medial (M) and superior (S) vestibular nuclei and in the prepositus hypoglosi nucleus (PH). Insets are showing the locations in the brainstem where the photomicrographs have been taken. Adapted from Ventre and Faugier-Grimaud (1988).

So far, if self-motion encoding seems to emerge from the activity of the superior temporal cortex, very few studies report on a possible top-down regulation by this region on visuo-vestibular-related structures. As described above, Dursteler and Wurtz (1988) have shown that chemical lesions in MST produce deficits in OKN induced by large visual field displacement. Otherwise, possible top-down influences are suggested by the existence of projections from the parieto-temporal cortex to the subcortical vestibular complex in monkey (Ventre and Faugier-Grimaud, 1988). Interestingly, by analyzing the topography of the injection sites in the parieto-temporal cortex, MST might have been encroached upon by our injections including the postero-ventral part of the parietal cortex and the dorsal banks of the superior temporal sulcus (Faugier-Grimaud and Ventre, 1989). As illustrated in **Figure 4** these parieto-temporal cortical sites were found to be directly projecting onto the vestibular nuclei complex including the medial vestibular and PH nucleus involved in the velocity storage integrator and gaze holding processing (Cheron et al., 1986a,b; Cannon and Robinson, 1987; Ventre and Faugier-Grimaud, 1988; Faugier-Grimaud and Ventre, 1989; Yokota et al., 1992). Interestingly, the connections from some extrastriate visual areas including MST responsible for visual motion processing in the far periphery might mediate rapid-response related information for orienting and postural reactions (Palmer and Rosa, 2006).

As a whole, the extrastriate areas including the middle superior temporal area (MST) are clearly implicated in processing movement information during ego-motion. In order to compensate for body displacements, the MST region in complement to other visual-vestibular cortical sites will preferentially compute velocity signals issued from the retina or head sensors and consequently it might contribute to eye/head coordination during rapid body orientation. This temporal cortical pole related to self-motion perception and rapid eye/head coordination might form a dissociated path from the parietal cortical pole whose implication in inertial vestibular signal integration and space representation will be considered in the following section. There, we will try to identify and assemble the functional features of this parieto-temporal region suggestive of integration (e.g., velocity to position) of visual and vestibular signals in the context of space representation.

## IS THE PARIETAL CORTEX IMPLICATED IN AN INERTIAL PROCESSING PATHWAY?

### IDENTIFICATION OF A VESTIBULAR PARIETO-TEMPORAL NETWORK

The first evidence of a pure vestibular cortical field was provided by evoked potentials techniques in the cat (Walzl and Mountcastle, 1949). These authors showed that a neuronal activity could be evoked by vestibular nerve stimulation in an anterior area of the suprasylvian sulcus, anterior to the auditory area and to the motion analysis suprasylvian cortex previously described. These observations in animal were in line with a temporal lobe hypothesis of a vestibular cortex suggested in human patients after cortical damage (Carmichael et al., 1954) or after electrical stimulation (Penfield and Jasper, 1954) of the temporal lobe. By electrophysiological recordings in the cat and in the monkey (Kornhuber and Da Fonseca, 1964; Fredrickson et al., 1966, 1974) single units were identified in the somatic area 2v that respond to caloric or galvanic labyrinthine stimulation.

A clear description of vestibular cortical fields were provided in behaving animals in the early 1980s by Mergner (1979) in the cat anterior suprasylvian cortex and by Kawano and Sasaki (1984), Kawano et al. (1980, 1984) in the macaque parietal associative cortex. Thus these electrophysiological recording studies provided evidence that vestibular inputs were integrated in cortex during sinusoidal rotation (Becker et al., 1979; Kawano et al., 1980). In Java monkey, Grusser et al. (1990a,b) identified neurons activated preferentially during angular acceleration in a separate region localized in the parieto-insular cortex. The authors called this region the parieto-insular vestibular cortex (PIVC) which they demonstrated further to be directly connected to the vestibular nuclei complex in the brainstem (Akbarian et al., 1994). At the same time, behavioral studies on the effects of focalized lesions in the parietal cortex confirmed Kawano’s observations (Kawano et al., 1980). Indeed, by using a unilateral cortical lesion approach (Ventre and Faugier-Grimaud, 1986), we demonstrated that unilateral damage of the postero-lateral part of area 7 in monkey induced vestibulo-ocular disturbances similar to those observed after a unilateral damage of the cortical homolog in the cat, the middle suprasylvian cortex (Ventre, 1985b). Such top-down effects on vestibulo-oculomotor function were confirmed as we found direct projections from this parietal cortex (previously lesioned: Ventre and Faugier-Grimaud, 1986) onto the vestibular nuclei complex as well as on the prepositus hypoglossi nucleus (NPH) in monkey (Ventre and Faugier-Grimaud, 1988, Faugier-Grimaud and Ventre, 1989).

**FIGURE 5:**
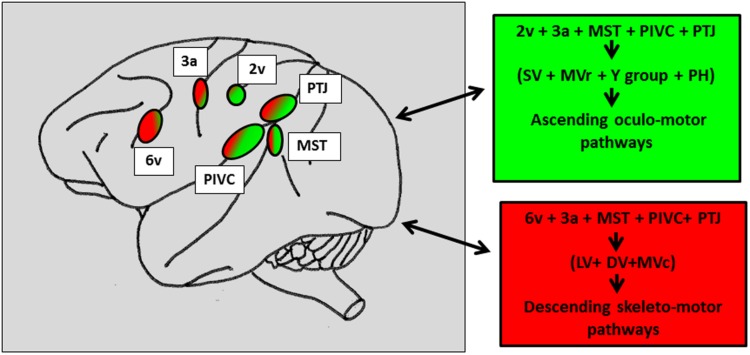
**Schematic representation of the vestibular cortical sites directly connected to the vestibular nuclei and the prepositus hypoglossi in macaque brain.** In the right panels: Projections topographically organized with the caudal vestibular cortical fields including MST, RI, and TPJ projecting more in the vestibular and prepositus hypoglossi nuclei, involved in the oculomotor ascending pathways (green patches) as compared to the more rostral cortical fields connected to the vestibular nuclei mainly involved in the skeletto-motor descending pathways (red patches). The numbers refer to the architectonically defined Brodman’s cortical areas. PTJ, Parieto-temporal junction; MST, middle superior temporal area; PIVC, parieto-insular vestibular cortex; Superior (SV), medio-rostral (MVr), medio-caudal (MVc), lateral (LV) and descending (DV) vestibular nuclei. Prepositus hypoglossi (PH) nucleus. Reconstructed from previous findings from Ventre and Faugier-Grimaud (1988), Akbarian et al. (1994).

The existence of a large network of cortical areas projecting onto the vestibular nuclei was also demonstrated by further anatomical works in monkey (Faugier-Grimaud and Ventre, 1989; Akbarian et al., 1993, 1994; Guldin et al., 1993). **Figure 5** synthesizes the different findings related to cortical projections onto the vestibular nuclei complex and prepositus hypoglossi distinguishing ascending oculomotor projections from descending skeletto-motor projections. Therefore, in the posterior associative cortex of monkey, evidence was provided for two distinct sites: (1) a caudal site corresponding to the posterior part of area 7 and (2) a second more rostral site, in the retro insular cortex corresponding to the so-called PIVC. In humans, a number of neuroimaging studies (PET and fMRI) investigated the cortical activation induced by caloric or galvanic stimulation of healthy subjects (Bottini et al., 1994, 2001; Bucher et al., 1997; Lobel et al., 1998; Bense et al., 2001; Fasold et al., 2002; Dieterich et al., 2003; Emri et al., 2003; Stephan et al., 2005; Eickhoff et al., 2006; Lopez et al., 2012). A neural network similar to the one described in monkey was found to be distributed between the parietal, temporal cortex and prefrontal cortices. While the postero-lateral part of the monkey parietal cortex is likely to correspond to the parieto-temporal cortex including area 39–40 on the parietal convexity (Ventre and Faugier-Grimaud, 1986, Faugier-Grimaud and Ventre, 1989; Kahane et al., 2003; Ventre-Dominey et al., 2003), the human analog of PIVC described in the posterior end of the insula in monkey remains quite uncertain. A number of neuro-imaging studies have suggested that the PIVC could correspond to the retro-insular cortex in humans (Dieterich and Brandt, 2001; Fasold et al., 2002; Stephan et al., 2005). However, based on correlations between functional imaging and cytoarchitectonic data, such an analogy has been recently revisited (Eickhoff et al., 2006; Zu Eulenburg et al., 2012) as the authors have localized in the posterior parietal operculum- the OP2 area- a putative human PIVC region (Grusser et al., 1990a,b). Accordingly, by using electrical stimulation in epilepsy patients, Kahane et al. (2003) could induce vestibular sensations by stimulating the temporo-parietal cortex close to the parietal operculum and less so the retroinsular cortex. Consequently, in the following we will refer to the parietal operculum i.e., the OP2 area, as the human cortical homolog of PIVC. At this point of our knowledge related to the organization of the vestibular cortex in primate, a question can be raised: whether these two parietal cortex (on the cortical surface) vs. parietal operculum- OP2- (in the sulcal depth) sites are functionally distinct or belong to a common cortical parietal network involved in vestibular function? So far, even though numerous studies including human investigations confirm the parietal involvement in vestibular function, the exact functional contribution of its different fields (TPJ vs OP2) remains unclear.

### VESTIBULAR INTEGRATION IN THE PARIETO-TEMPORAL NETWORK AND SPATIAL PROCESSES

#### Representation of the extrapersonal space

**FIGURE 6:**
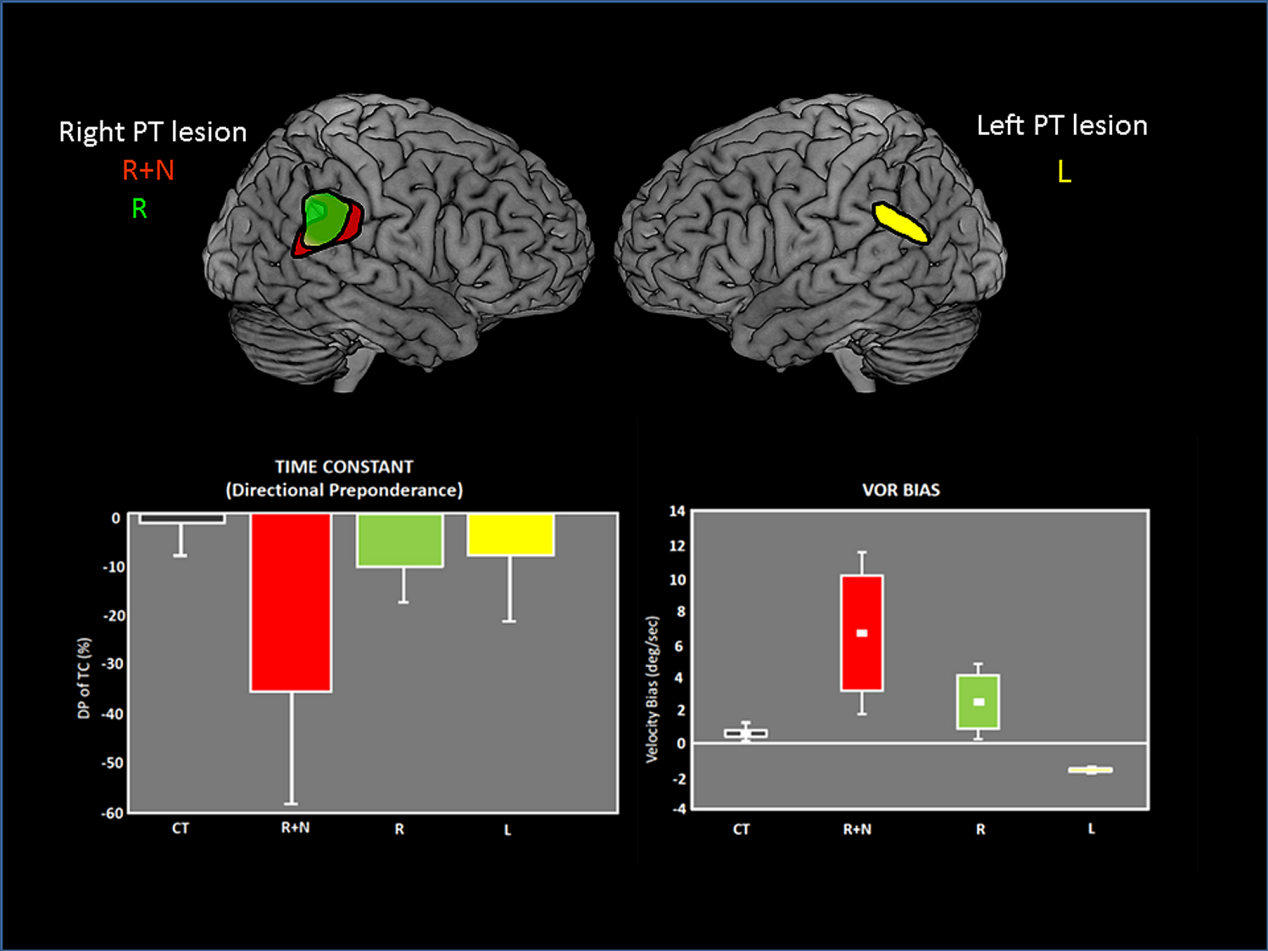
**Vestibulo-ocular (VOR) deficits observed in patients after unilateral lesions in the parieto-temporal junction.** On the top, the common lesioned sites have been reconstructed and are represented for each group of patients on the lateral views of brain templates: R+N: right lesion with neglect. R: right lesion without neglect. L: Left lesion. On the bottom, graphs showing the asymmetrical deficits in VOR time constant (measured by the directional preponderance) and the VOR bias in each patient group. The deficits are majored with right lesions with neglect. Adapted from Ventre-Dominey et al. (2003).

The temporo-parietal cortical junction constitutes a major hub of multi-sensory convergence and transformations underlying spatial representation. Such a role of the parietal cortex in spatially oriented behavior has been revealed by the neglect syndrome that can develop in patients after right parietal damage (for review, Karnath and Dieterich, 2006; Karnath and Rorden, 2012). A visuo-spatial neglect is a neurological disorder characterized by a difficulty for patients with a right brain injury to respond or orient themselves to persons or objects located in the contralesional space. Neglect patients usually exhibit spontaneous and sustained deviation of their eyes and head toward the side of the brain damage. Even though neglect syndrome was often linked to right parietal damage, structural brain imaging demonstrates the involvement of a perisylvian cortical network including the parieto-temporal junction, the inferior parietal lobe, the superior/middle temporal cortex and the ventrolateral prefrontal cortex. Interestingly this cortical perisylvian network partially overlaps with the temporo-peri-Sylvian vestibular network defined by Kahane et al. (2003) on the basis of vestibular symptoms evoked in a large group of epileptic patients. This peri-Sylvian vestibular network would preferentially process vestibular canal signals that transduce angular head accelerations (Kahane et al., 2003). The similarity in the topographical aspects of the neglect vs vestibular peri-sylvian networks is also paralleled with a similarity in behavioral deficits, i.e., ipsilesional eye and head deviation observed in neglect patients as well as in patients with peripheral vestibular loss. Furthermore, it has been shown that a durable compensation of the neurological spatial disorders can occur after unilateral labyrinthine stimulation in neglect patients (Rubens, 1985; Vallar et al., 1995). In keeping with this idea of a linkage between parieto-temporal cortex, visuo-spatial, and vestibular functions, we have shown vestibulo-ocular deficits in patients with unilateral parieto-temporal lesions (Ventre-Dominey et al., 2003). As illustrated in **Figure 6**, the vestibular deficits were preeminent for the inertial components of the vestibulo-ocular reflex (the time constant and bias) and were significantly linked to visuo-spatial disorders (Ventre-Dominey et al., 1999, 2003). Accordingly, by using a novel paradigm combining bistable perceptual stimuli or complex attentional tasks with concurrent vestibular stimulation in healthy human subjects, Arshad et al. (2013) report similar findings suggestive of a top-down cortical regulation of the VOR time constant. In a patient with a residual neglect consecutive to an occipito-parieto-temporal damage, the ability to update the contralesional visual space after angular rotation was perturbed (Ventre-Dominey and Vallee, 2007). In normal subjects, Seemungal et al. (2008), Seemungal (2014) found that repetitive transcranial magnetic stimulation (rTMS) of the right parietal cortex (comparable to a virtual lesion) disrupted the perceptual encoding of vestibularly driven displacement in contralateral space. On the basis of these observations we infer that the parieto-temporal cortex exerts a down regulation of the vestibulo-ocular function in particular the inertial (low frequency) component implying the velocity storage integrator.

Evidence for the top-down influence of the parieto-temporal cortex in primate is strengthened by the existence of direct anatomical pathways connecting this part of the posterior cortex to the vestibular nuclei complex (**Figure 4**). Indeed, we have demonstrated in monkey that the parietal cortex located posteriorly to the PIVC projects directly onto the vestibular nuclei including the medial vestibular and PH nuclei involved in the velocity storage integrator and gaze holding processing (Cheron et al., 1986a,b; Cannon and Robinson, 1987; Ventre and Faugier-Grimaud, 1988; Faugier-Grimaud and Ventre, 1989; Yokota et al., 1992). Based on our findings and those of the recent literature, we suggest that the parietal cortex constitutes a unique cortical region involved in high-ordered multimodal transformation of inertial vestibular signals including the updating of the visual space during body displacements.

#### Representation of self-referential space

The idea of a vestibular influence in space representation involving the parieto-temporal cortex has been extended to self-referential processing by a series of experiments using illusory percepts (Blanke and Arzy, 2005; Tsakiris and Haggard, 2005; Ionta et al., 2011a,b, Lopez and Blanke, 2011). For example, the most commonly described experiment the so-called rubber-hand illusion demonstrates how a sensory conflict between visual and tactile signals can produce changes in bodily self-referential (Tsakiris and Haggard, 2005). In the rubber-hand illusion, the participant is looking at a stroked rubber hand while his/her own hidden hand is synchronously stroked. After a delay, the participant reports the sensation of his/her own hand to be positioned close to the fake hand, or even of an illusory ownership of it.

**FIGURE 7:**
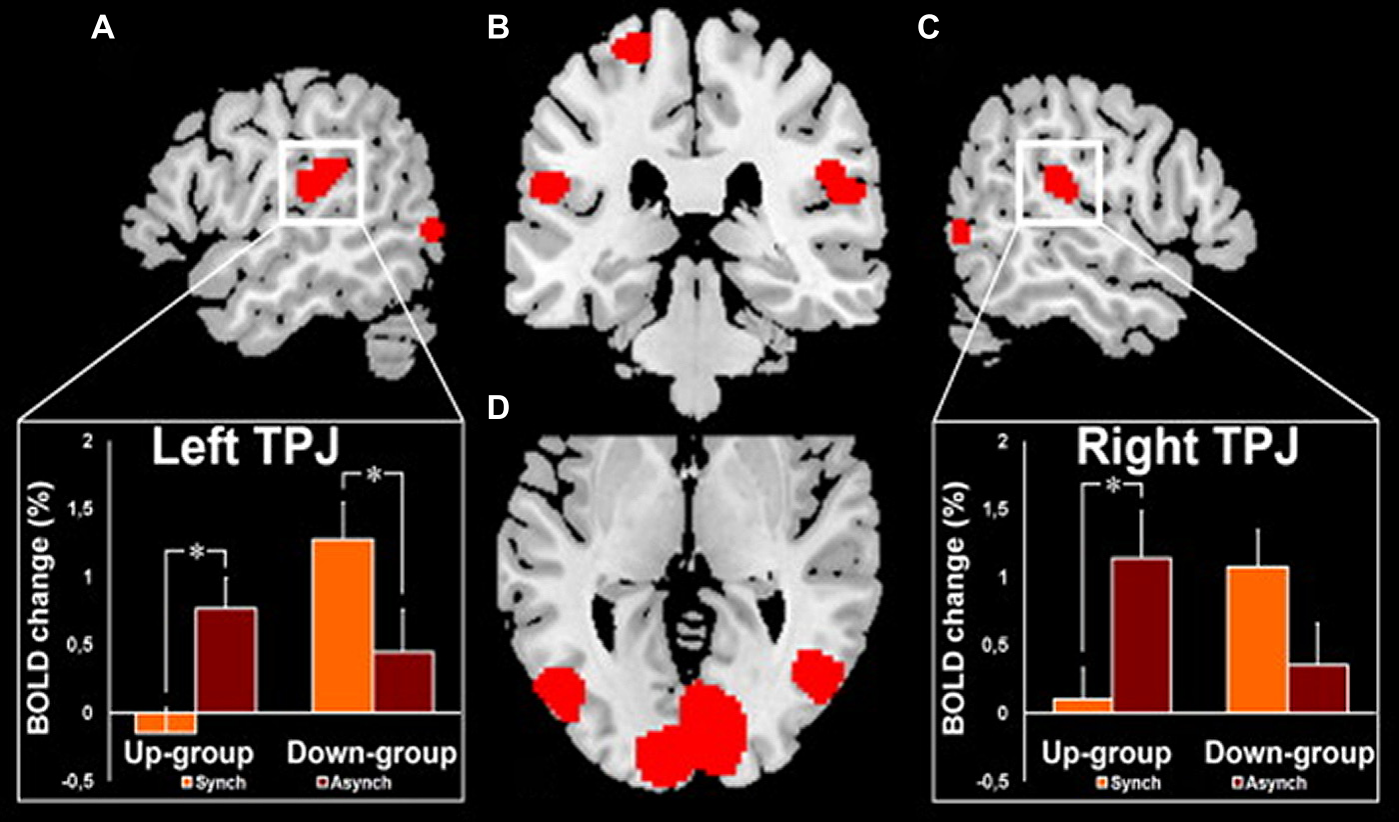
**fMRI activation in the right and left temporo-parietal junctions (TPJ) in healthy subjects during experimentally induced out of body experience (OBE).** In the Up-group, the subjects experience themselves to look upward at a visually presented body and to be spatially higher with the synchronous as compared to the asynchronous strocking of their back. In the Down-group, the opposite pattern of sensations is observed as the subjects experience themselves to look downward and to be spatially higher with the asynchronous as compared to the synchronous strocking of their back. The magnitude of the blood- oxygenation-level-dependent (BOLD) responses in the TPJ is lower in the condition of higher self-location in the synchronous and in the asynchronous strocking, respectively, for Up-group and for Down-group. **(A)** Left TPJ activation with inset showing the corresponding BOLD changes in each group and condition. **(B)** Activation in the left and right TPJ and in the left superior postcentral gyrus. **(C)** Right TPJ activation with inset showing the corresponding BOLD changes in each group and condition. **(D)** Bilateral activation of the posterior middle and inferior temporal gyri. *Significant differences and bars: standard errors. Reproduced from Ionta et al. (2011b).

Interestingly, such an illusory perception of a false hand ownership is emphasized by galvanic vestibular stimulation using the same paradigm of the rubber-hand illusion (Lopez et al., 2010). Lopez et al. (2010) concluded that such a vestibular interference is mediated by the parieto-temporal and posterior insular cortex. In the same vein, clinical observations of out-of-body experiences (OBEs) suggest that localized cortical lesions can induce pathological changes of first-person perspective and self-location in space (Blanke et al., 2002, 2004; Blanke and Mohr, 2005; De Ridder et al., 2007). Similarly to the rubber-hand illusion, the OBE has been reproduced in healthy subjects by manipulating tactile and visual information on the back of their body (Ehrsson, 2007; Lenggenhager et al., 2007). In this experiment, by synchronously scrubbing the participant’s back and the back of a visually displayed virtual body, changes in self-consciousness occur as the participant experiences a drift of the first person perspective and the self-location toward the virtual body. In a recent neuroimaging study, this same group (Ionta et al., 2011b) demonstrates that the illusory shift in bodily self-referential was reflected in changes in cerebral activation of the parieto-temporal cortex (TPJ) contiguous to the cortical area within the TPJ that is lesioned in OBE patients. As shown in **Figure 7**, these authors reported two kinds of OBE sensations: – in the Up-group, the subjects experiences themselves to be looking upward at the visually displayed body and estimated their self-location to be higher during a synchronous as compared to an asynchronous stroking of their back and – in the Down-group, the subject’s experiences themselves to be looking downward at the visually displayed body and estimated their self-location to be higher during asynchronous as compared to synchronous stroking as some subjects. The TPJ blood-oxygenation-level-dependent (BOLD) response decreased in the Up-group only during the synchronous condition and the opposite for the Down-group with a decreased BOLD TPJ response in the asynchronous condition. Ionta et al. (2011b) suggest that the TPJ activity reflects drift-related changes in self-location within each group that depend differently on the experienced direction of the first-person perspective. The OBE percept has been linked to interactions between gravitational visual and vestibular cues which might take place in TPJ (Ionta et al., 2011b). Thus OBE might rely on the remapping of self-location in extra-personal space based on a double disintegration of bodily visual and vestibular signals taking place in the TPJ and the nearby posterior parietal operculum. These recent observations related to bodily self-consciousness stress the importance of the vestibular function in high-order multimodal processing taking place in the posterior cortex including the temporo-parietal junction possibly including the posterior parietal operculum.

## CONCLUSION AND THEORETICAL PERSPECTIVES

**FIGURE 8:**
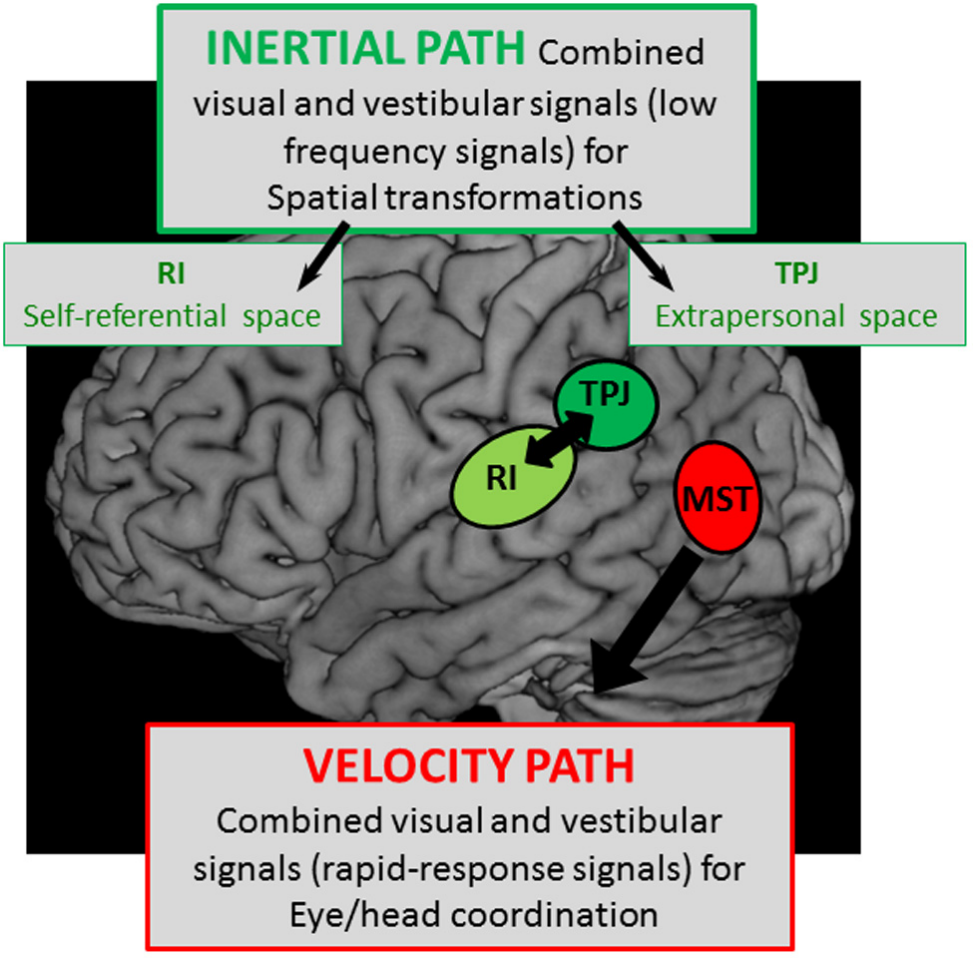
**Schematic representation of two hypothetical cortical pathways with – a velocity path including the middle superior temporal cortex (MST) where visual and vestibular signals are mediated to serve for rapid-responses during eye/head coordination, and – an integration pathway of visual and vestibular signals with low kinematic (low frequencies) in the temporo-parietal junction (TPJ) and the retro-insular cortex (RI) possibly involved in the construction respectively of the extrapersonal and self-referential spaces**.

In conclusion, this review on the different components of the lateral parietal and temporal cortex leads to the idea of two separate cortical vestibular fields as it is schematically represented in **Figure 8**:

The first involves the extrastriate temporal area including MST which receives well-defined visual and vestibular velocity signals likely involved in heading perception. On the basis of the oculomotor kinetic-related properties and the lesion effects of this cortical region, it is likely that MST mediates both visual and vestibular information in order to compensate for head motion thereby contributing to eye/head coordination. Such a rapid top-down regulation of visuo-vestibular interactions might be subtended by direct descending pathways from MST to vestibular nuclei and prepositus hypoglossi involved in gaze control.

The second involves the parietal cortex including parieto-temporal junction and posterior parietal operculum, both implicated in high-order multimodal integration and cognitive functions, including peri-personal space and self-referential processing. In this context, the vestibular information would be processed in the parietal cortex in connection with the subcortical vestibular nuclei complex for a velocity storage integration that might contribute to the construction of spatial reference frames. Such an integration pathway might be responsible: (1) for extrapersonal space transformations preferentially in the parieto-temporal junction subtending visuo-spatial orientation and (2) for self-referential processing involving the bodily information about self-location in space that might be mediated preferentially by the parieto-opercular pole of this local vestibular parietal network.

These posterior vestibular fields are likely part of a more extended cortical network, such as the peri-sylvian network described above and implicated in high-order cortical processes linked to spatial referential processing. Moreover, if such a hypothetical cortico-vestibular architecture involved in cognitive functions has been built on the basis of the literature review, this hypothesis forms the basis for a program of future research.

## Conflict of Interest Statement

The author declares that the research was conducted in the absence of any commercial or financial relationships that could be construed as a potential conflict of interest.
